# Retirement Savings Model Tested With Brazilian Private Health Care Workers

**DOI:** 10.3389/fpsyg.2019.01701

**Published:** 2019-07-23

**Authors:** Thais C. Schuabb, Lucia H. França, Silvia M. Amorim

**Affiliations:** Department of Psychology, Salgado de Oliveira University, Niterói, Brazil

**Keywords:** retirement, financial planning, goals, savings, well-being

## Abstract

Retirement is one of the most serious challenges facing Brazil currently, considering the rapid pace of population aging, growing social inequalities, and the difficulty that Brazilians have in planning for their financial future. The number of studies on psychosocial factors in retirement is limited. The aim of this cross-sectional study is to determine Brazilian health workers’ perceptions about financial planning for retirement, based on studies by Hershey and Mowen (2000). In this study, retirement saving – the dependent variable - is highlighted by the use of a model with the following antecedents: parental influence, retirement goal clarity and retirement planning activity level. The goals of the study were to establish mediating and moderating relationships as an innovative approach to the original model. Data was gathered from 319 workers at a private hospital in the municipality of Niterói, Rio de Janeiro (Brazil) who filled out a questionnaire concerning their saving behaviors and antecedents. Results indicated a model in which goal clarity mediated the relationship between parental influence and retirement saving, and retirement activity was observed to influence the level of retirement saving. The findings confirmed the complexities of financial planning for retirement, and emphasized important factors, such as parental advice starting in childhood and the effect of this advice on goal clarity. The results also pointed to the role of individual responsibility in the process, which depended on establishing a plan for activities. In addition to parental advice, an educational approach can contribute encouraging saving behaviors and helping retirees achieve financial security in retirement.

## Introduction

Currently, population aging represents one of the most serious challenges. The growth of this older population has resulted in an exponential number of retirees (World Health Organization [WHO], 2015). In Brazil, as in other developing countries, the aging process is occurring more quickly (Brazilian Institute of Geography and Statistics [IBGE], 2017). In 2040, the number of elderly Brazilian people is expected to constitute almost one fourth (23.8%) of the population (Miranda et al., 2016). For this reason, demographic change is a challenging issue, especially considering the economical-political crisis and the unsustainable public system of retirement pensions (Ataides and Santos, 2017; Garcia and Haro, 2017). Therefore, retirement planning has generated a number of discussions in many sectors throughout Brazil.

From a psychological point of view, retirement can be a time of satisfaction or stress, depending on retirement-related behaviors developed over the course of the years preceding retirement (França and Vaughan, 2008). Recent studies have confirmed the importance of retirement planning in different aspects such as finance, health, social activity, and psychology (França and Soares, 2009; Zanelli et al., 2010; Yeung, 2013; Boehs et al., 2018).

Previous studies have reported that achieving well-being and satisfaction in retirement depends on several factors, such as financial safety, good health, satisfactory interpersonal relationships, engaging in leisure and work activities (Gutierrez and Hershey, 2014; Muratore and Earl, 2014; Amorim et al., 2017; Guerson et al., 2018). Among these factors, financial safety appears to be the most critical aspect to be achieved in retirement projects in order to contribute to personal satisfaction in this stage of life (Petkoska and Earl, 2009; França and Hershey, 2018; Seidl et al., 2018).

In Brazil, individuals often start working early in life. The National Household Sample Survey (Pesquisa Nacional por Amostra de Domicílios - PNAD) revealed that more than 70% of active workers started working before the age of 17 (Brazilian Institute of Geography and Statistics [IBGE], 2015), suggesting that for young people, entering the job market may not be a planned decision. This data also suggests that leaving the job market is also not planned, and perhaps this is why most workers decide to continue working even after retirement. In a study conducted by the Hong Kong and Shanghai Banking Corporation (HSBC) in 17 countries, Brazil is of one the three countries where active workers plan least for retirement and leaving the job market (Hong Kong and Shanghai Banking Corporation [HSBC], 2015). A majority of Brazilian workers (62%) report that they continue working full or part time, whereas globally, only 56% of older workers do so. In addition, less than one third (28%) of Brazilian workers plan to be totally retired upon leaving the job market (compared to the global mean of 34%). A total of 10% of Brazilians state that they do not plan to retire at all (Hong Kong and Shanghai Banking Corporation [HSBC], 2015).

The Retirement Preparation Programs (RPP) was established in Brazil to support workers in their retirement decisions, in parallel to incentives for voluntary early retirement programs (França and Soares, 2009). Three decades later, these programs are more concerned with promoting retirees’ well-being and have been recommended by Brazilian law (Brazil, 2003). Despite this, a survey of managers from 207 organizations (França et al., 2014) showed that although a majority of Brazilian managers consider such a program important, only one quarter of organizations have adopted the program. Around half of Brazilian managers feel that the program should be offered at least three to 5 years before retirement (França et al., 2014).

In many aspects, including financial, emotional, social and family relationships, such programs have the potential to impact the quality of life for a number of future retirees (Leandro-França et al., 2016). In general, organizations adopt these programs 1–2 years before workers are scheduled to retire, and sessions include lectures and workshops to promote discussion about health, retirement legislation, economic issues in retirement, family relationships, volunteering and leisure, among others topics (Zanelli et al., 2010). RRP sessions often last 24 h or more, and they aim to help attendees build a new life project (França and Vaughan, 2008; França and Soares, 2009; Leandro-França et al., 2016).

The lack of adequate retirement planning is a serious concern, both economically and politically, as well as individually. Lack of adequate financial planning for retirement may negatively affect retirees who may end up lacking sufficient income in this final stage of life (Heraty and McCarthy, 2015; Sartori et al., 2016). Helping workers anticipate the risk of income reduction requires an understanding of the planning process, clearly defined reasons for when and why people retire, how they prepare for this stage of life, and how they adjust their lives for retirement (Hershey and Mowen, 2000; Hershey et al., 2007b, 2010). Because of the importance of financial planning for retirement and the number of factors that can influence planning for retirement, Hershey and Mowen (2000) have proposed a psycho-motivational model of financial planning for retirement, in which the dependent variable represents the saving behavior in retirement, i.e., individual practices of saving for retirement. They describe how such behavior is influenced by psychological, social, economic and cultural variables (Hershey and Mowen, 2000).

The Hershey and Mowen (2000) model sought to identify the relationship between knowledge of financial planning and characteristics of individuals’ personality (future perspectives, emotional stability, conscientiousness and relevance and impact of retirement) during planning for retirement. The authors reported direct and indirect influences between the constructors investigated, and they concluded that both personal structure and financial knowledge are important predictors for adequate financial planning for retirement (Hershey and Mowen, 2000).

Other studies attempted to refine Hershey and Mowen’s model by improving understanding of the aspects that influence financial planning for retirement (Hershey et al., 2010; Gutierrez and Hershey, 2014; Koposko and Hershey, 2014). In addition, studies conducted in a variety of countries on this topic used many variables considered in the psycho-motivational model by Hershey and Mowen (2000), and they included new variables such as parental influence and education in childhood (Koposko and Hershey, 2014; Topa et al., 2018), the influence of friends (Koposko et al., 2016), clarity of retirement goals (Stawski et al., 2007; Topa et al., 2018), financial risk tolerance (Jacobs-Lawson and Hershey, 2005), trust in government pension (França and Hershey, 2018) and planning activity level (Hershey et al., 2007b).

As stated by Kerry (2018), the practice of saving for retirement has already been addressed in different studies including sociodemographic factors such as income, marital status and gender (Petkoska and Earl, 2009; Gutierrez and Hershey, 2014), cognitive antecedents, i.e., financial literacy and financial knowledge (Jacobs-Lawson and Hershey, 2005; Lusardi and Mitchell, 2007), cognitive antecedents, i.e., financial risk tolerance, retirement goal clarity and future time perspective (Stawski et al., 2007; Koposko and Hershey, 2014; Earl et al., 2015; Koposko et al., 2016), and affective antecedents, i.e., emotional stability, neuroticism and extroversion (Hershey and Mowen, 2000; Kerry and Embretson, 2018).

Although these studies constitute important advances for understanding relations among these variables and how this model functions in different cultures, a number of gaps exist to define a comprehensive model of planning for retirement. Only one study was found that addressed the Brazilian context. In that study, França and Hershey (2018) replicated the psycho-motivational model developed by Hershey and colleagues to investigate psychological factors that influence Brazilians’ financial planning for retirement. The study was the first step to understanding this process from a psychological perspective. These authors found that future time perspective and retirement goal clarity were the main predictors of adequate perception of saving. The authors emphasized, however, the importance of new Brazilian studies mainly related to financial planning for retirement, as well as research to reveal psychological aspects that can predict and influence this planning (França and Hershey, 2018).

The results found to date support the conclusion that financial planning for retirement is extremely important and should be encouraged among workers as early as possible. The importance of this factor is corroborated by studies from the field of psychology (Hershey and Mowen, 2000; Topa et al., 2011). Although many workers understand the importance of financial planning in retirement decisions, this emerging topic needs further research, actions and policies in the organizational and governmental area. Data presented above reinforces the relevance of the present study, which aims to examine the views of health care professionals with regard to financial planning for retirement, considering psychological, social and economic factors.

**FIGURE 1:**
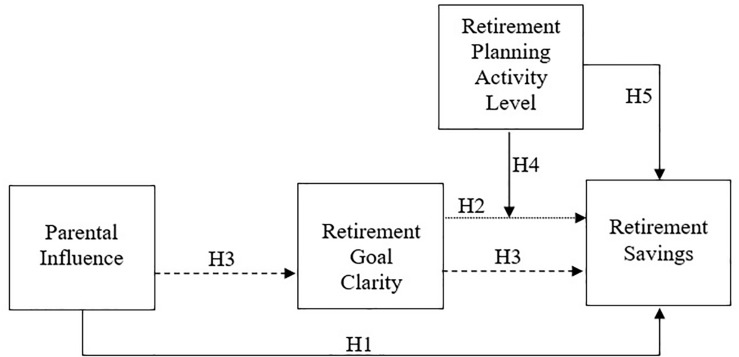
Financial Planning for Retirement Model developed by the authors based on Hershey and contributors (Hershey and Mowen, 2000; Hershey et al., 2007a, 2010; Stawski et al., 2007; Koposko and Hershey, 2014; Koposko et al., 2016; França and Hershey, 2018).

Considering the theoretical references analyzed, a cross sectional study model (Figure 1) was used to test and explain saving behavior, based on the following antecedents: parental influence on saving habits, retirement goal clarity and retirement planning activity level. These three antecedents for financial planning for retirement were chosen from among other factors identified as influencing this process for specific reasons. Parental influence and retirement goal clarity were selected as possible variables that can be accessed and developed from external interventions. The same is not the case for income, gender, and age related variables, which cannot be changed by the researcher (Stawski et al., 2007; Koposko and Hershey, 2014). Retirement planning activity level was also selected as an antecedent of financial planning for retirement because of the positive relationship between this factor and saving for retirement found in previous studies (Hershey et al., 2007a, b, 2010; Stawski et al., 2007; França and Hershey, 2018).

### Parental Influence and Retirement Savings

Parental influence on the lives of their children has been studied in several spheres. This influence is also found in financial planning for retirement, considering that acquisition of financial skills often begins with education on income management offered by parents (Grinstein-Weiss et al., 2012). Parental influence is therefore a measure used to quantify the effect that parents have on individual money management skills and on saving habits (Palaci et al., 2017). This is an important variable to be considered, mainly for the motivational force of enhancement, different from variable such as income and personality traits that are harder to change through external efforts (Koposko and Hershey, 2014).

In a study of Brazilian retirees, França and Hershey (2018) found a direct relationship between early life learning experiences and financial knowledge deriving from such experiences. These results suggest that, in Brazil, lessons about financial knowledge from parents early in life have a considerable and direct impact on individuals’ financial knowledge. As financial knowledge has already been directly associated with level of activities undertaken in the direction of retirement planning, and this latter is directly related to retirement savings (Hershey et al., 2007a, 2010), parental lessons seem also to influence retirement savings. Based on these results, the first hypothesis was formulated:

Hypothesis 1: Parental influence has a direct and positive relation with retirement savings.

### Retirement Goal Clarity and Retirement Savings

Goals stimulate planning and help to anticipate the future, support the prior construction of experience perceptions, and allow individuals to set expectations about needs they will have when facing specific times (Petkoska and Earl, 2009). In the case of retirement, goals also play an important role. A clear retirement goal involves planning, and this requires effective savings actions (Stawski et al., 2007). This construct involves the act of thinking, discussing and establishing future goals, specifically those related with quality of life in retirement (Hershey et al., 2007b).

Previous studies verified that retirement goal clarity acts as antecedent of two main constructors: level of activity planning and financial knowledge (Hershey et al., 2007a,b; Stawski et al., 2007; Koposko and Hershey, 2014; Koposko et al., 2016). These two constructors, however, act as predictors for retirement saving, retirement contributions or saving adequacy. Based on these results, a relationship between retirement goal clarity and retirement savings was hypothesized. In addition, studies have identified that parental influence acts as an antecedent of retirement goal clarity (Hershey et al., 2010; Koposko and Hershey, 2014). Understanding that retirement goal clarity acts as antecedent of retirement savings and consequence of parental influence, it was possible to hypothesize a mediator role of this variable.

Hypothesis 2: Retirement goal clarity has a positive and direct relationship with retirement savings;
Hypothesis 3: Retirement goal clarity mediates the relationship between parental influence and retirement savings.

### Activities of Planning and Retirement Savings

The relationship between planning behaviors and personal savings practices has been confirmed for decades (Lusardi, 1999). As expected, the level of planning activity for retirement has already been confirmed to have a direct association with retirement savings, both in the perception of saving adequacy (Hershey et al., 2007b, 2010; França and Hershey, 2018) and effective savings contributions (Hershey et al., 2007a; Stawski et al., 2007). For this reason, planning activity is a measurement responsible for assessing the frequency of both information-seeking and effective planning activities that have occurred in the last 12 months (Hershey et al., 2010).

These activities involve a number of behaviors that stimulate knowledge about investments, and consequently, savings practices. Among these behaviors, those that we can emphasize are: searching for information, participating in lectures about the subject, and attending a retirement planning program (Stawski et al., 2007). Considering previous results that planning activity level was identified as a consequence of retirement goal clarity and antecedent of retirement savings (Hershey et al., 2007a,b, 2010; Stawski et al., 2007), a positive and direct relation and a moderating role of this variable were hypothesized.

Hypothesis 4: Planning activity level moderates the relationship between retirement goal clarity and retirement savings.
Hypothesis 5: Planning activity level has a direct and positive relation on the level of retirement savings.

## Materials and Methods

### Participants

A total of 319 workers from the technical, treatment and administrative areas of a private hospital in the city of Niterói, Rio de Janeiro, Brazil, participated in this quantitative study. In this convenience sample, health workers were the selected population based on several criteria: (i) health workers have special rights for retirement (Brazil, 1991); (ii) there is a higher rate of occupational diseases among health workers, such as burnout syndrome (Moore and Cooper, 1996); (iii) health workers have greater ability to choose an autonomous career, which presupposes a different work regimen than that established for hired employees (Zoltowski and Teixeira, 2013).

The sample consisted of women with a mean age of 36, less than half of whom were married or in a stable relationship. The level of formal education of the sample was higher than Brazil as a whole, with most of participants having earned high school or higher degrees. The main area of work was nursing. Workload was high, between 37 and 45 h weekly, but their earning did not correspond to the level of education and workload, as the average earnings were up to four times the minimum wage. Their family incomes were, on average, ten times the minimum wage, and this income was shared with one or two dependents (Table 1).

**TABLE 1:** Sociodemographic description of the sample.

**Variable**	***N***	***M* (SD)**	**%**
Age (in years)	319	36.2 (8.33)	
20–30	95		29.6
31–40	138		43.3
41–50	64		20.3
51–60	22		6.8
**Gender**			
Male	81		25.4
Female	238		74.6
Marital Status			
Married/In a stable relationship	174		54.5
Single/Divorced/Widowed	145		45.5
**Occupational area**			
Nursing	152		47.6
Administration^*^	89		27.9
Rehabilitation	16		5.0
Radiology	14		4.4
Pharmacy, Nutrition, Medicine	20		6.4
Other	28		8.8
**Education**			
Elementary school (complete/incomplete)	11		3.4
High school/technical (complete/incomplete)	107		33.6
Higher education (complete/incomplete)	117		36.7
Post-graduation (complete/incomplete)	84		26.3
**Workload**			
Up to 24 h a week	20		6.3
Between 25 and 36 h a week	61		19.1
Between 37 and 45 h a week	169		53
More than 45 h a week	69		21.6
**Monthly personal income (R$)**			
Up to 2 min. wages (up to R$ 1,908.00)	154		48.3
From 2 to 4 min. wages (R$ 1,908.01 to R$ 3,816.00)	107		33.5
From 4 to 10 min. wages (R$ 3,816.01 to R$ 9,540.00)	52		16.3
Above 10 min. wages (R$ 9,540.01)	6		1.9
**Monthly family income (R$)**			
Up to 2 min. wages (up to R$ 1,908.00)	51		16
From 2 to 4 min. wages (R$ 1,908.01 to R$ 3,816.00)	125		39.2
From 4 to 10 min. wages (R$ 3,816.01 to R$ 9,540.00)	106		33.2
Above 10 min. wages (R$ 9,540.01 to R$ 19,080.00)	37		11.6
**Number of dependents**	319	2.98 (1.17)	
No dependents	35		11
1 or 2 dependents	182		56.9
3 or 4 dependents	96		30.1
More than 5 dependents	6		1.9

Min., Minimum; Max., Maximum. ^*^Administration, Administration, Finance, Maintenance Engineering, Reception, Human Resources, and General Services.

### Instruments

The questionnaire was designed using scales already applied in previous studies which tested the Hershey and Mowen (2000). The items were translated from English into Brazilian Portuguese, and the instrument was also semantically adapted to the Brazilian context. Translation into Brazilian Portuguese was conducted by two English-language teachers, one a retired public school English teacher and the other an English teacher currently teaching at a Brazilian federal university. The instrument was also revised by a Brazilian specialist in this field fluent in English.

#### Retirement Savings

The instrument measured individual retirement saving practices (Jacobs-Lawson and Hershey, 2005). It included five items, (i.e., “I make a conscious effort to save for retirement.”). A Likert-type response format was used (1 = strongly disagree; 7 = strongly agree). Other studies found significant results, presenting a good Cronbach’s alpha coefficient: 0.93 (Jacobs-Lawson and Hershey, 2005) and 0.79 (Hershey et al., 2010). In our sample, the Cronbach’s alpha coefficient was 0.92 (M = 2.37; SD = 0.96).

#### Parental Influences on Saving

The instrument measured the effect of parental influence on money management and on creating a saving habit among their children (Koposko and Hershey, 2014). The instrument included six items (i.e., “Saving money for the future was an important lesson I learned as a child.”). This was a Likert-type response format (1 = strongly disagree, 7 = strongly agree). Other studies found significant results, presenting a good Cronbach’s alpha coefficient: 0.78 and 0.77 (Gutierrez and Hershey, 2014) and 0.86 (Koposko and Hershey, 2014). In our sample, the Cronbach’s alpha coefficient was 0.87 (M = 3.09; SD = 0.97).

#### Retirement Goal Clarity

The instrument included an assessment of the act of thinking, discussing and setting future goals, specifically those related to quality of life in retirement (Hershey et al., 2007b). It included five items, (i.e., “I set up clear goals to become informed about retirement.”). This was a Likert-type response format (1 = strongly disagree; 7 = strongly agree). Other studies found significant results, presenting a good Cronbach’s alpha coefficient: 0.87 (Hershey et al., 2007b) and 0.87 (Koposko and Hershey, 2014). In our sample, the Cronbach’s alpha coefficient was 0.83 (M = 2.95; SD = 0.84).

#### Retirement Planning Activity Level

The instrument measured both the frequency of searching for information and the level of effective planning activities that had occurred within the last 12 months (Hershey et al., 2010). It included four items (i.e., “I have informed myself about financial preparation for retirement.”). This was a Likert-type response format (1 = strongly disagree, 7 = strongly agree). Other studies found significant results, presenting a good Cronbach’s alpha coefficient: 0.84 (França and Hershey, 2018) and 0.84 (Hershey et al., 2010). In our sample, the Cronbach’s alpha coefficient was 0.90 (M = 2.35; SD = 0.96).

### Procedures

#### Data Collection

Questionnaires were made available in print to control shipment and facilitate the calculation of the response rate. The following strategies were used to gather results from 319 participants. First, the principal researcher contacted a large private hospital in the municipality of Niterói to introduce the project to the Human Resources coordinator; then, permission was requested to conduct the research within the institution. After receiving approval, the researcher remained in the hospital for several days to interview the employees in their workplace. Participants were interviewed using a printed and self-applied questionnaire distributed during their lunch time, as employees usually have lunch in the hospital). This approach was effective and guaranteed a fair degree of participants from the private sector, with a response rate of 87%.

#### Data Analysis

The database was analyzed to categorize variables and verify missing and outlier cases. Because it was the first application of the instrument in the Brazilian context, it was decided to verify the structure of the instruments and identify the best model. A confirmatory factor analysis (CFA) was therefore conducted.

To verify the existence of multicollinearity between latent variable, a Pearson correlation was used, and correlations were classified as low (between 0.10 and 0.29), moderate (0.30 and 0.49) and high (higher than 0.50), as suggested by Miles and Shevlin (2001). For a testing model, a structural modeling analysis was used for step-by-step analysis of the paths, starting from the direct effect between the independent variable and the dependent variable, and including the mediator and moderator variables using a variety of models (Vieira, 2009).

Analysis was performed using MPlus software version 6. The maximum likelihood estimator (ML) was used to maximize the probability that data were taken from the population. The adjusted chi-square, CFI, TLI, GFI, RMSEA and SRMR indexes were evaluated according to Byrne’s (2001) recommendations related to well-adjusted models. In addition, the following criteria were considered evidence of satisfactory adjustment: CFI values close to 0.90, GFI close to 0.90, TLI close to 0.90 and RMSEA and SRMR close to or less than 0.08 (Byrne, 2001).

#### Ethical Procedures

This study was approved by the Ethics Committee of the Universidade Salgado de Oliveira on February 22, 2018, under number CAAE 82650018.3.0000.5289. Prior to completing questionnaires, the participants signed the consent term, confirming their willingness to participate in the study. In this consent form, participants were also informed that their responses would be treated as confidential and that they were free to leave the study at any time.

## Results

### Descriptive Statistics

The sample included 319 workers (238 women, 81 men) between 20 and 60 years of age, with a mean age of 36. The distribution of professionals by working area included a majority from nursing area (47.6%), followed by administration (18.2%). Remaining areas had low percentages, as described in Table 1.

The level of formal education ranged from incomplete elementary school to graduate studies. Workload varied from 25 to 45 h per week. Personal income was, on average, two to four times the Brazilian minimum wage. The majority of participants (66.5%) did not have a private pension or savings for retirement. The majority of participants were married or had stable relationships (54.5%), while the remainder (45.5%) were single, divorced or widowed.

### Confirmatory Factorial Analysis

To confirm or exclude the factorial structure of the instrument created by Hershey and colleagues (Jacobs-Lawson and Hershey, 2005; Hershey et al., 2007a,b; Gutierrez and Hershey, 2014; Koposko and Hershey, 2014), we performed a CFA of the Retirement Saving, Parental Influence, Retirement Goal Clarity, and Retirement Planning Activity Level scales. The four instruments confirmed the unidimensional structure established by their creators, with good indexes of adjustment (CFI > 0.96, TL I > 0.92, RMSEA < 0.14, SRMR < 0.03), as observed in Table 2. All items were maintained in the instruments, with factorial loads above 0.60, as shown in Table 3, which presents the alpha, means and standard deviations of scales, their items, and their respective factorial loads.

**TABLE 2:** Adjust indexes and adjust of factorial load of items of the scales (*n* = 319).

**Adjust indexes**	**χ2 (gl)**	**CFI**	**TLI**	**RMSEA**	**SRMR**
Retirement Saving Scale (RS)	36,478(5)	0.97	0.95	0.14	0.02
Parental Influences on Saving Scale (PI)	48,461(9)	0.96	0.93	0.12	0.03
Retirement Goal Clarity Scale (GC)	29,449(5)	0.96	0.92	0.12	0.03
Retirement Planning Activity Level Scale (PA)	5,972(2)	0.99	0.99	0.08	0.01

**TABLE 3:** Descriptive data (Factorial loads, means, standard deviation, alpha).

	**F1**	**M**	**SD**
**Retirement Saving Scale (RS) (α = 0.92)**		2.37	0.96
I have made meaningful contributions to a voluntary retirement savings plan.	0.77	2.31	1.10
Relative to my peers, I have saved a great deal for retirement.	0.85	2.41	1.10
I have accumulated substantial for retirement.	0.88	2.29	1.05
I have made a conscious effort to save for retirement.	0.89	2.41	1.09
Based on how I plan to live my life in retirement, I have saved accordingly.	0.83	2.44	1.12
**Parental Influences on Saving Scale (PI) (α = 0.87)**		3.09	0.97
Saving money for the future was an important lesson I learned as a child.	0.62	3.17	1.26
My parents did a good job of planning and saving for their own retirement.	0.71	2.70	1.18
My parents would expect me to save for retirement.	0.74	3.08	1.26
Growing up, my parents helped me to imagine situations when I might need extra money to fall back on.	0.74	2.93	1.26
My parents made sure that I understood the value of money and that money is a limited resource.	0.79	3.48	1.23
My parents suggested to me concrete ways to save money on my own.	0.81	3.18	1.19
**Retirement Goal Clarity Scale (GC) (α = 0.83)**		2.95	0.84
I set clear goals for gaining information about retirement.	0.77	2.89	1.03
I have thought a great deal about quality of life in retirement.	0.68	3.41	1.10
I set specific goals for how much will need to be save for retirement.	0.80	2.67	1.04
I have clear vision of how life will be in retirement.	0.72	2.80	1.05
I have discussed retirement plans with spouse, friend or significant other.	0.61	3.00	1.22
**Retirement Planning Activity Level Scale (PA) (α = 0.90)**		2.35	0.96
Calculations have been made to estimate how much I have to save to retire comfortably.	0.72	2.33	1.08
I frequently read books, brochures, or surf the web to learn about retirement planning.	0.80	2.20	1.06
I have informed myself about the level of future pension benefits.	0.88	2.52	1.16
I have informed myself about financial preparation for retirement.	0.93	2.35	1.08

### Model Testing

Initially, an existing correlation between latent variables was observed and it was noticed that VME values were, in the majority, higher than coefficient of determination (*r*^2^) between latent variables (i.e., VME > *r*^2^). These results indicated the lack of multicollinearity, with the exception of multicollinearity found between Planning Activity Level and Retirement Saving, and Planning Activity Level and Goal Clarity (Table 4). Next, the influence of gender variables, marital status, age and family income on the dependent variable was observed. Considering that the age and the family income had an influence on retirement savings, they were controlled in the models described below.

**TABLE 4:** Correlation between latent variables (below diagonal) and coefficient of determination (above diagonal) (*n* = 319).

	**VME**	**RS**	**PI**	**GC**	**PA**
Retirement Saving Scale (RS)	0.71		0.16	0.49	0.56
Parental Influences on Saving Scale (PI)	0.49	0.40^*^		0.12	0.06
Retirement Goal Clarity Scale (GC)	0.51	0.70^*^	0.35^*^		0.55
Retirement Planning Activity Level Scale (PA)	0.70	0.75^*^	0.24^*^	0.74^*^	

^*^p < 0.05.

First, a model that did not include the mediator or moderator variables was tested to verify the effect of parental influence on retirement savings (Hypothesis 1), followed by a model with the goal clarity level as a mediator (Hypothesis 2 and 3). To test hypothesis 4, one moderated-mediation model was tested. In this model, goal clarity mediated the relationship between parental influence and retirement savings, and planning activity was a moderator of the relationship between parental influence and goal clarity. Results showed that moderation was not significant (β = 0.10, *p* = 0.09).

Finally, a fourth model was checked, considering the goal clarity level as a mediator of the relationship between parental influence and retirement saving, and the direct effect of planning activity on retirement savings (Hypothesis 5). This model presents the mediation effect and a significant effect of this variable on the dependent variable (β = 0.19). All of these models can be observed in Table 5.

**TABLE 5:** Non-standardized coefficient Effects of interaction between variables in four models (*n* = 319).

****	**RS on PI [β(SE)]**	**RS on GC [β(SE)]**	**GC on PI [β(SE)]**	PA on GC^*^PA [β(SE)]	**RS on PA [β(SE)]**
Model 1	0.452(0.085)^*^	–	–	–	–
Model 2	0.210(0.063)^*^	0.665(0.092)^*^	0.347(0.074)^*^	–	–
Model 3	0.212(0.054)^*^	0.287(0.067)^*^	0.374(0.070)^*^	0.093(0.055)^ns^	–
Model 4	0.219(0.059)^*^	0.280(0.078)^*^	0.344(0.073)^*^	–	0.591^*^(0.086)

*RS, Retirement Saving; PI, Parental Influence; GC, Goal Clarity; PA, Planning Activity. *p* < 0.001; ^*ns*^ non significance.*

**FIGURE 2:**
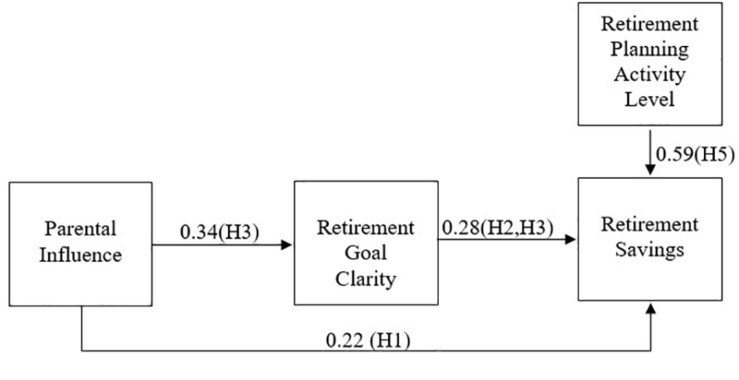
Financial Planning for Retirement Model with found effects (β).

These data allow us to conclude the existence of a partial mediation of the relationship between parental influence and retirement savings, since the significant effect among the variables was reduced when the variable goal clarity was included in the model. In addition, planning activity represents a strong effect on retirement saving. This model presented good adjustment indices, despite the high residual value, [χ2 (gl) = 605.127 (165); CFI = 0.896; TLI = 0.880; RMSEA = 0.092; SRMR = 0.144]. Hypotheses 1, 2, 3, and 5 were thus confirmed. The final model is described in Figure 2.

## Discussion

This study investigated health workers in order to understand their perception about financial planning for retirement, as well as how these are related to psychological, social and economic factors involved this process. A study model (Figure 1) sought to explain retirement savings behaviors based on three antecedents: parental influence, retirement goal clarity, and retirement planning activity level.

It was observed (Table 3) that means found in total score ranged from 2 and 3, and that the sample studied was within or below the mean, with regard to financial planning for retirement. Low means obtained in scales can be interpreted as indicating a significant lack of knowledge about financial planning among Brazilians. This assessment may be skewed by the mean age of the participants (M = 36.2; SD = 8.33). Because the participants were relatively young, they may not have given much thought to retirement.

However, there is no doubt that more knowledge is needed with regard to Brazilians’ financial planning for retirement. The more this topic is studied, the clearer the picture becomes regarding the Brazilian population’s familiarity with financial planning. These results support existing demands for development of more strategic actions on this topic (França and Hershey, 2018). Further study is also needed among public sector workers to compare behavior changes with respect to financial planning for retirement between workers in the public and private sectors and the invariance of this model.

It is generally known that financial planning for retirement is a complex process, in which several factors play an important role in predicting and constructing effective savings behaviors for this stage of life. As a whole, the scale chosen to evaluate the dependent variable (retirement saving behaviors) as well as the three antecedents (parental influence, retirement goal clarity and planning activity level) revealed good psychometric indices, which is consistent with results found in previous studies that adopted the same scales (Jacobs-Lawson and Hershey, 2005; Hershey et al., 2007b; Stawski et al., 2007; Hershey et al., 2010; Gutierrez and Hershey, 2014; Koposko and Hershey, 2014; Koposko et al., 2016; França and Hershey, 2018).

Our first hypothesis was confirmed, since parental influence had a significant direct effect on the level of savings for retirement, before other variables were included in the model. The habit of saving, originated in the family, influences the construction of attitudes and behaviors over the course of a person’s lifetime. For this reason, it seems that parents have a crucial role in this process (Grinstein-Weiss et al., 2012). The confirmation of this hypothesis, therefore, attests that significant parental influence increases the chances that an individual will save for retirement (Gutierrez and Hershey, 2014; Koposko and Hershey, 2014; Koposko et al., 2016). Since this is a more likely antecedent to be accessed and improved, the confirmation of parental influence on financial skills and retirement savings, actions and strategies is paramount for financially responsible behavior in preparation for retirement (Koposko and Hershey, 2014; Palaci et al., 2017; França and Hershey, 2018).

Our second hypothesis was also confirmed, in that goal clarity represented a positive influence on retirement savings. Retirement goal clarity is an important psychological predictor of retirement planning that is developed during adulthood and motivates the individual to plan for the future (Stawski et al., 2007; Petkoska and Earl, 2009). This relationship has been observed in previous studies and is thus reinforced by the Brazilian context: greater retirement goal clarity is associated with more effective retirement savings behaviors (Koposko and Hershey, 2014; França and Hershey, 2018). This result was considered important because these goals are not fixed characteristics of the personality, but they can be developed with interventions aimed at planning for retirement (Hershey et al., 2007a; Petkoska and Earl, 2009; Koposko et al., 2016).

In addition to the positive influences found by both parental influence (Koposko and Hershey, 2014) and goal clarity (Stawski et al., 2007) with regard to retirement savings, it was also observed that clarity of goals mediated the relationship between parental influence and retirement savings. This confirmed our third hypothesis. This relationship means that the effect of parental influence on savings for retirement diminished when goal clarity was included in the model, and it better explains the dependent variable. This result can be interpreted as the probable parental influence on the creation of specific goals for retirement (Koposko et al., 2016) to influence saving behavior.

On the other hand, the fourth hypothesis, related to the moderating effect of the level of planning activity between goal clarity and retirement savings, was not confirmed. In contrast to the findings reported in a previous study (Stawski et al., 2007), in which goal clarity had an indirect influence on retirement savings through planning activities, our study could not confirm this relationship.

Considering that the planning activity level represented an influence on the level of retirement savings, the fifth hypothesis was confirmed. The degree to which an individual engages in retirement planning activities has a direct and positive relationship on the individual’s adoption of a retirement saving behavior. As reported in previous studies, the adoption of planning behaviors is associated with higher levels of personal saving practice (Lusardi, 1999; Stawski et al., 2007; França and Hershey, 2018). In psycho-motivational models that were already tested, the antecedents exercise both a direct and indirect influence on the final saving behavior for retirement. Among these relationships, the retirement planning activity level correlates with retirement saving (Stawski et al., 2007; Hershey et al., 2010; França and Hershey, 2018). It is possible to conclude that by engaging in retirement planning activities, an individual performs the first planning step and then tends to continue with saving behavior in an effective way.

The results of this study describe important contributions related to Brazilian behaviors regarding financial planning for retirement. Findings have reported the importance of financial planning, which should start as early as possible. Financial necessity is one of the factors that prevent people from retiring, in addition to the financial aspects be considered one of the main losses in retirement. Therefore, financial planning has a role in making retirement possible, reducing its perception of loss (Petkoska and Earl, 2009; Heraty and McCarthy, 2015).

The role of government as the sole source of financial support during a citizen’s retirement has been increasingly questioned worldwide. Consequently, this responsibility to guarantee comfort in this stage of life is now transferred to the individual, which reinforces the importance of our study by broadening the understanding of this complex phenomenon to establish financial planning for retirement (Yusof and Sabri, 2017).

In addition to the results already found in the previous Brazilian study on financial planning for retirement (França and Hershey, 2018), our findings show an untested mediating relationship in this process. All of the relationships found indicate that parents play a crucial role in encouraging saving behaviors. Our findings also highlight the issue concerning individual responsibility and the behavior required to achieve a desired level of financial comfort during retirement. These aspects can guide interventions to educate people, raise awareness, reinforce and reverse their behaviors with regard to financial planning for retirement (Gutierrez and Hershey, 2014; Koposko and Hershey, 2014). Intercultural and cross-cultural studies are warranted to observe whether the mediating and moderating functions can also be confirmed in other contexts.

Our study has some limitations. First, the sample was limited to workers in a single private health care facility in the municipality of Niterói, Rio de Janeiro, Brazil. Therefore, the results cannot be extrapolated to other population outside Brazil. The fact that the majority of the sample was composed of workers aged 20 to 40 years is another limitation, since older workers tend to be more involved with planning for retirement especially in Brazil, where there is no culture of long-term planning (França et al., 2017). Despite this, the few existing studies, generally performed with older workers or retired people, demonstrate the low level of preparation even when retirement is approaching (França and Vaughan, 2008; França and Soares, 2009).

The existence of other antecedents responsible for influencing the complex process of financial planning for retirement needs to be emphasized. In future studies, the new relationships of variables can be tested. In addition, other populations should be investigated, considering that our study included only health workers. Our hypotheses highlight the need to better understand the role of planning activities in the model studied. Longitudinal studies are also recommended to measure the effectiveness of actions designed to develop skills related to financial planning for retirement.

The lack of depth of psychological understanding of the reasons related to developing a financial plan for retirement opens up a range of potential studies in this subject. Because of the complexity of issues involved in retirement planning, simply promoting a lecture or a short workshop is not sufficient for people to understand how to manage their investments, especially when they are approaching retirement.

Retirement Preparation Programs constitute an attempt to help workers to develop life projects, and these programs are supported by the recommendation of the Brazilian Statute of the Elderly (Brazil, 2003). In addition, financial literacy is beginning to gain ground in schools, mainly after the implementation of the National Financial Education Strategy (ENEF) (Brazil, 2010), which may help to introduce financial planning behavior early in people’s lives. To contribute to such initiatives, studies, especially longitudinal ones, are warranted to identify factors that influence the adoption of financial planning for retirement. All these efforts can offer suggestions for future interventions and improvements to existing ones.

We expect that the results of this study can help improve our understanding of factors that influence Brazilians workers from the private health sector with regard to their financial planning for retirement. Strategies and interventions must be created to improve and even reverse the current lack of planning for retirement in Brazil, which seems to be a common behavior requiring immediate action (Triandis, 1995; Hofstede et al., 2010). This initiative also expects to inspire more studies on this topic, including workers from the public sector and other areas with the aim of extending this important and relevant topic in our society.

## Ethics Statement

This study was carried out in accordance with the recommendations of Health National Council - CNS, no. 466 and the Ethical Committee of University Salgado de Oliveira with written informed consent from all subjects. All subjects gave written informed consent in accordance with the Declaration of Helsinki. The protocol was approved by the University Salgado de Oliveira Committee under the number 82650018.3.0000.5289.

## Author Contributions

TS designed the study and collected the data. LF designed the study. All authors analyzed the data and wrote the manuscript.

## Conflict of Interest Statement

The authors declare that the research was conducted in the absence of any commercial or financial relationships that could be construed as a potential conflict of interest.
